# A Novel Biological Approach to Treat Chondromalacia Patellae

**DOI:** 10.1371/journal.pone.0064569

**Published:** 2013-05-20

**Authors:** Jaewoo Pak, Jung Hun Lee, Sang Hee Lee

**Affiliations:** 1 Stems Medical Clinic, Seoul, Republic of Korea; 2 National Leading Research Laboratory, Department of Biological Sciences, Myongji University, Yongin, Gyeonggido, Republic of Korea; Rush University Medical Center, United States of America

## Abstract

Mesenchymal stem cells from several sources (bone marrow, synovial tissue, cord blood, and adipose tissue) can differentiate into variable parts (bones, cartilage, muscle, and adipose tissue), representing a promising new therapy in regenerative medicine. In animal models, mesenchymal stem cells have been used successfully to regenerate cartilage and bones. However, there have been no follow-up studies on humans treated with adipose-tissue-derived stem cells (ADSCs) for the chondromalacia patellae. To obtain ADSCs, lipoaspirates were obtained from lower abdominal subcutaneous adipose tissue. The stromal vascular fraction was separated from the lipoaspirates by centrifugation after treatment with collagenase. The stem-cell-containing stromal vascular fraction was mixed with calcium chloride-activated platelet rich plasma and hyaluronic acid, and this ADSCs mixture was then injected under ultrasonic guidance into the retro-patellar joints of all three patients. Patients were subjected to pre- and post-treatment magnetic resonance imaging (MRI) scans. Pre- and post-treatment subjective pain scores and physical therapy assessments measured clinical changes. One month after the injection of autologous ADSCs, each patient's pain improved 50–70%. Three months after the treatment, the patients' pain improved 80–90%. The pain improvement persisted over 1 year, confirmed by telephone follow ups. Also, all three patients did not report any serious side effects. The repeated magnetic resonance imaging scans at three months showed improvement of the damaged tissues (softened cartilages) on the patellae-femoral joints. In patients with chondromalacia patellae who have continuous anterior knee pain, percutaneous injection of autologous ADSCs may play an important role in the restoration of the damaged tissues (softened cartilages). Thus, ADSCs treatment presents a glimpse of a new promising, effective, safe, and non-surgical method of treatment for chondromalacia patellae.

## Introduction

Chondromalacia patellae (CMP), defined as cartilaginous softening and fibrillation of patellar bone cartilage, is one of the possible cause of patellofemoral pain syndrome (PFPS) [1]. PFPS is characterized by anterior knee pain (AKP) and accounts for 10–25% of all visits seen in physical therapy clinics [1]. CMP can be documented by magnetic resonance imaging (MRI) scans [2]. Currently, there is no definite cure for cartilaginous softening (e.g., CMP) thus presenting a major therapeutic challenge. However, a few recent studies have shown the possibility of cartilage recovery using mesenchymal stem cells (MSCs) [3], [4]. Further, from June 2009, the Korean Food and Drug Administration has allowed medical uses of non-expanded stem cells processed in a medical facility [5]. In this report, we describe the first successful approach to dramatic reduction of AKP in CMP by percutaneously injection of autologous adipose-tissue-derived stem cells (ADSCs: one kind of MSCs) along with platelet-rich plasma (PRP), 0.5% hyaluronic acid, and 3% CaCl_2_; a ADSCs mixture.

## Materials and Methods

This is a retrospective case series with the primary endpoint being safety and efficacy (pain improvement at month 1 and 3 along with MRI evidence of probable cartilage regeneration after 3 months) of ADSCs mixture-based treatment.

### Inclusion and exclusion criteria, and outcome endpoints

The inclusion criteria, exclusion criteria, and outcome endpoints are listed as follows. Inclusion criteria: (i) MRI evidence of chondromalacia patellae; (ii) either male or female; (iii) under 60 years of age; (iv) an unwillingness to proceed with surgical intervention; (v) the failure of conservative management; and (vi) ongoing disabling pain. Exclusion criteria: (i) active inflammatory or connective tissue disease thought to impact pain condition (i.e., lupus, rheumatoid arthritis, fibromyalgia); (ii) active endocrine disorder that might impact pain condition (i.e., hypothyroidism, diabetes); (iii) active neurological disorder that might impact pain condition (i.e., peripheral neuropathy, multiple sclerosis); and (iv) active pulmonary disease requiring medication usage. Outcome endpoints (obtained at three months after treatment): (i) pre- and post-treatment VAS (visual analog scale) walking index; (ii) pre- and post-treatment Functional Rating Index; (iii) pre- and post-treatment MRI; and (iv) telephone questionnaires every six months: at 6, 12, and 18 months.

### Pain score and physical therapy

Functional rating index (FRI) and visual analog scale (VAS) were determined as previously described [3]–[4], [6]–[7].

### Medication restrictions

Patients were restricted from taking steroids, aspirin, non-steroidal anti-inflammatory drugs (NSAIDs), and Asian herbal medications for one week prior to the procedure.

### Liposuction

In the operating room, approximately 40 mL of packed adipose tissue were obtained by liposuction of the subcutaneous layer of the lower abdominal area using sterile techniques [1], [8].

### Preparation of autologous ADSCs mixture

ADSCs were extracted through the use of digestive enzymes (0.07% type 1 collagenase; Adilase, Worthington, Lakewood, NJ, USA) and centrifugation (500 *g*) [4], [8]–[9]. The total volume of the solution containing ADSCs was 8.5 mL. While preparing the ADSCs, 30 mL autologous blood were drawn along with 2.5 mL anticoagulant citrate dextrose solution (0.8% citric acid, 0.22% sodium citrate, and 0.223% dextrose; Baxter Healthcare Corp., Marion, NC, USA). After centrifugation (100 *g*, then 1000 *g*), 4.4 mL of PRP along with the Buffy coat were obtained. 0.5% (w/v) hyaluronic acid (2 mL; Huons, Chungbuk, Korea) was added as a scaffold to this mixture, and 3% (w/v) CaCl_2_ (0.1 mL; Choongwae Pharmaceutical Co., Gyeonggido, Korea) was added to activate PRP. These ADSCs along with PRP, hyaluronic acid, and CaCl_2_ stand for the ADSCs mixture.

### ADSCs mixture-based treatment

After the left knee was cleaned with 5% povidone-iodine (Choongwae Pharmaceutical Co., Seoul, Korea) and draped in a sterile fashion, the injection site was anesthetized with 2% lidocaine (Daehan Pharmaceutical Co., Gyeonggido, Korea). The ADSCs mixture (15 mL) was injected into the retro-patellar joint on the day of liposuction with a 20-gauge, 1 1/2-inch needle under ultrasonic guidance. On the third and seventh day after the initial injection, another dose of PRP with CaCl_2_ and hyaluronic acid (1 mL) was injected as the same fashion as the first day. On the fourteenth day after the initial injection, a low-dose (254.8 nmol/L) dexamethasone (Huons, Chungbuk, Korea) was added to PRP with CaCl_2_. On day 28, the last dose of PRP with CaCl_2_ was injected.

### Patient characteristics

According to Korean law (Rules and Regulations of the Korean Food and Drug Administration), this study does not need approval by ethical and scientific committees [5].

Further, this study was approved and classified as exempt by the Solutions Institutional Review Board (Approval No. 1301260). After obtaining written informed consents from all the participants, Jaewoo Pak, M.D. made the clinical decisions on treatments.

**Figure 1:**
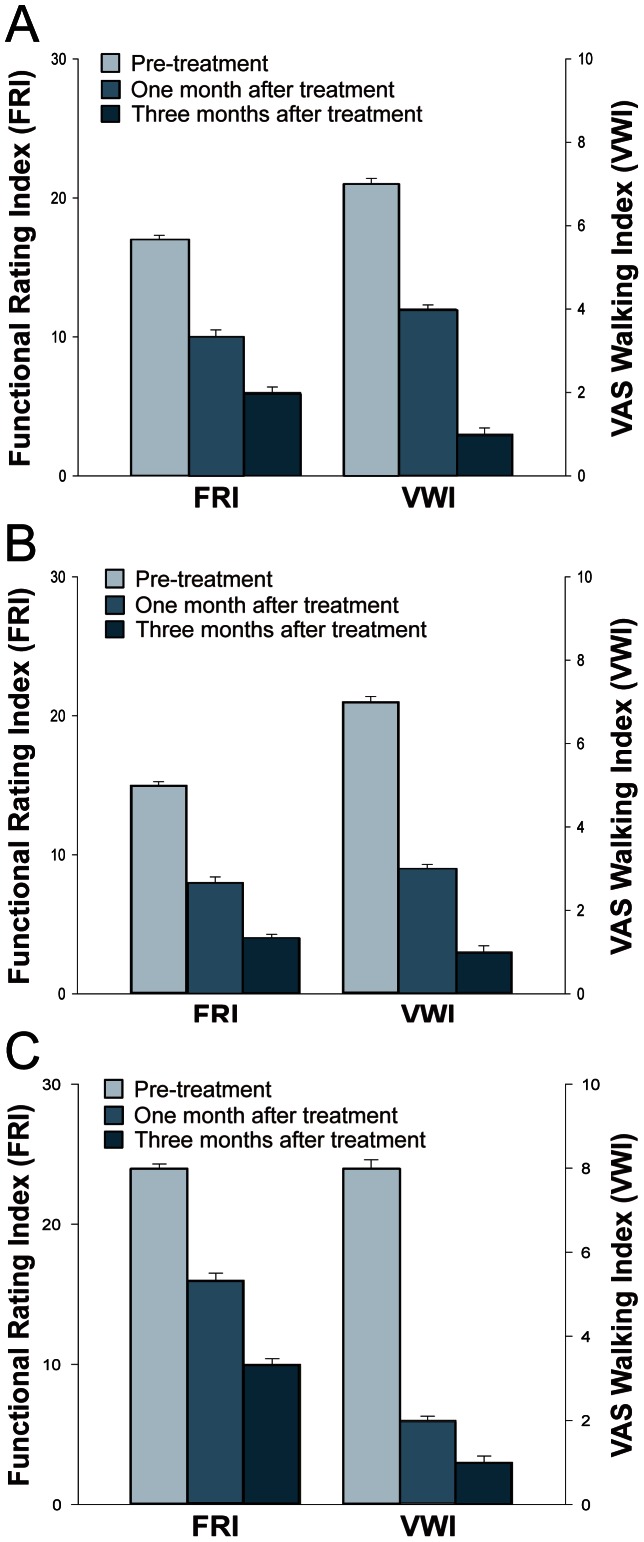
Pain measurements of patients 1 (A), 2 (B), and 3 (C). VAS is visual analog scale and T bars indicate standard deviations.

**Figure 2:**
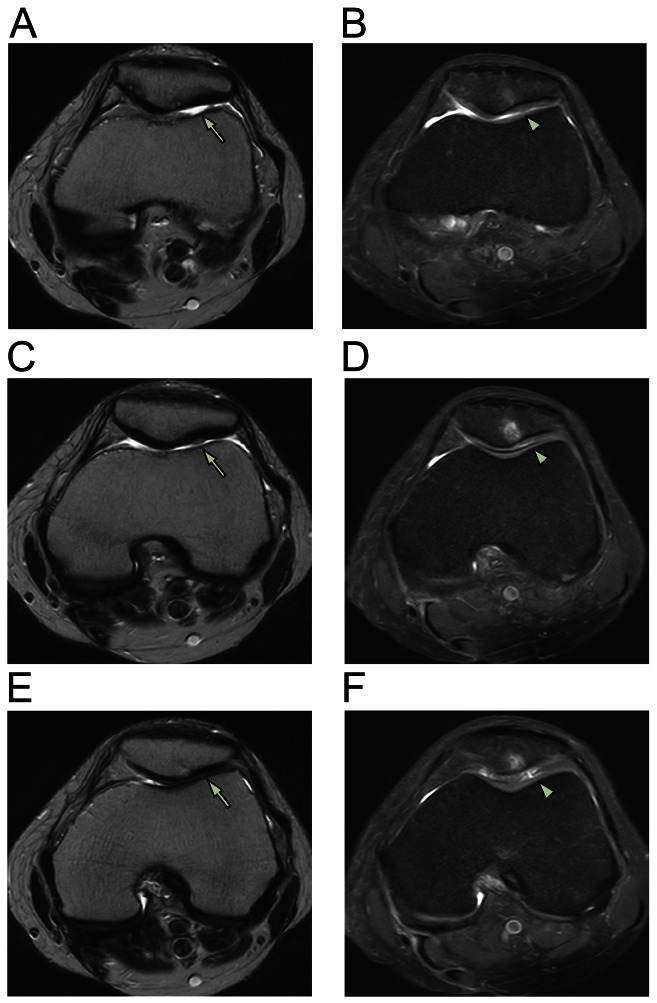
MRI axial sequential T2 views of patient 1. Pre-treatment MRI scans of patient 1 (A [sequential image: 3/16], C [4/16], and E [5/16]) show retro-patellar signal changes (arrow) consistent with chondromalacia patellae (upper bone). At three months, post-treatment MRI scans of patient 1 (B [6/20], D [7/20], and F [8/20]) show changes (arrowhead) consistent with probable cartilage restoration on the patellae-femoral joint.

The patient #1 is a 43-year-old Korean female with more than one year history of right AKP. The patient started having AKP without any history of trauma. She was seen by a physician and was diagnosed with chondromalacia of the knee after MRI imaging studies. After taking NSAIDs for few weeks along with physical therapy (PT), the AKP improved. However, approximately 6 months prior to our clinic visit, the patient started having AKP again. The pain was worse when standing up, walking, exercising, but improved with rest. The pain was not much relieved with NSAIDS and PT, this time. At the time of initial evaluation, the patient reported mild pain (VAS score: 2) on rest, increased pain when walking (VAS walking index [VWI]: 7). The FRI was 17 (Fig. 1A). The physical examination was non-remarkable except mild tenderness at the medial retro-patellar region. The Q-angle was 17. MRI scans showed retro-patellar signal changes consistent with CMP (Fig. 2A, C, and E).

**Figure 3:**
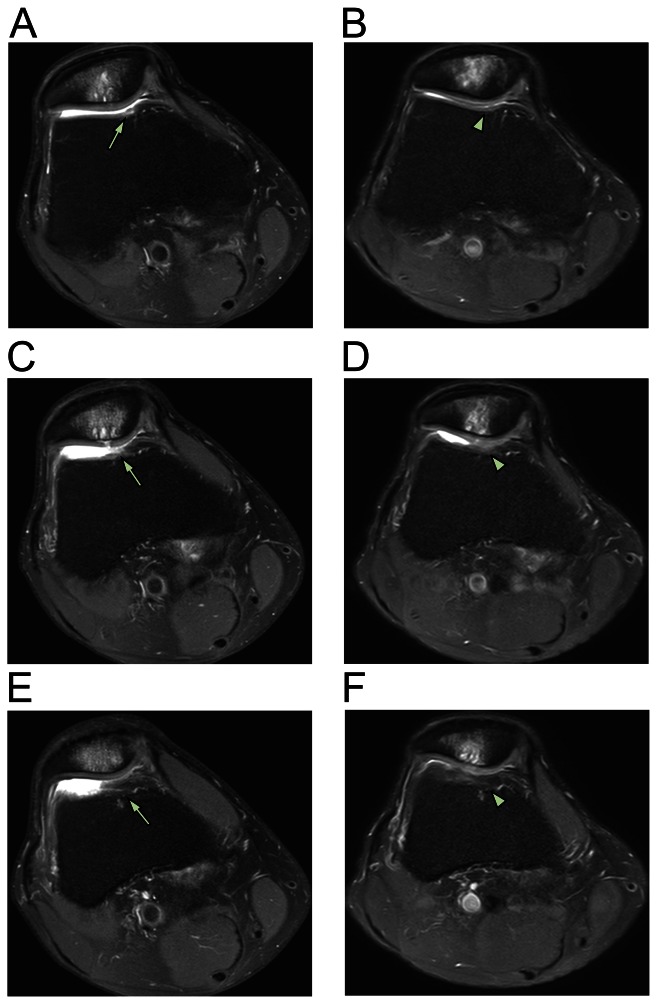
MRI axial sequential T2 views of patient 2. Pre-treatment MRI scans of patient 2 (A [16/24], C [17/24], E [18/24]) show retro-patellar signal changes (arrow) consistent with chondromalacia patellae (upper bone). At three months, post-treatment MRI scans of patient 2 (B [right; 4/20], D [5/20], and F [6/20]) show changes (arrowhead) consistent with probable cartilage restoration on the patellae-femoral joint.

The patient #2 is a 54-year-old Korean male with 3 to 4 year history of right AKP. The patient started having AKP without any history of trauma except vigorous uses of knees, such as mountain hiking. He was seen by a physician and was diagnosed with CMP of the knee after MRI imaging studies. The patient took NSAIDs along with PT, without much improvement. At the time of initial evaluation in our clinic, the patient reported mild AKP (VAS score: 2) on rest, increased pain when walking (VWI: 7). The FRI was 15 (Fig. 1B). The physical examination was non-remarkable except mild tenderness at the medial retro-patellar region. The Q-angle was 14. MRI scans showed retro-patellar signal changes consistent with CMP (Fig. 3A, C, and E).

**Figure 4:**
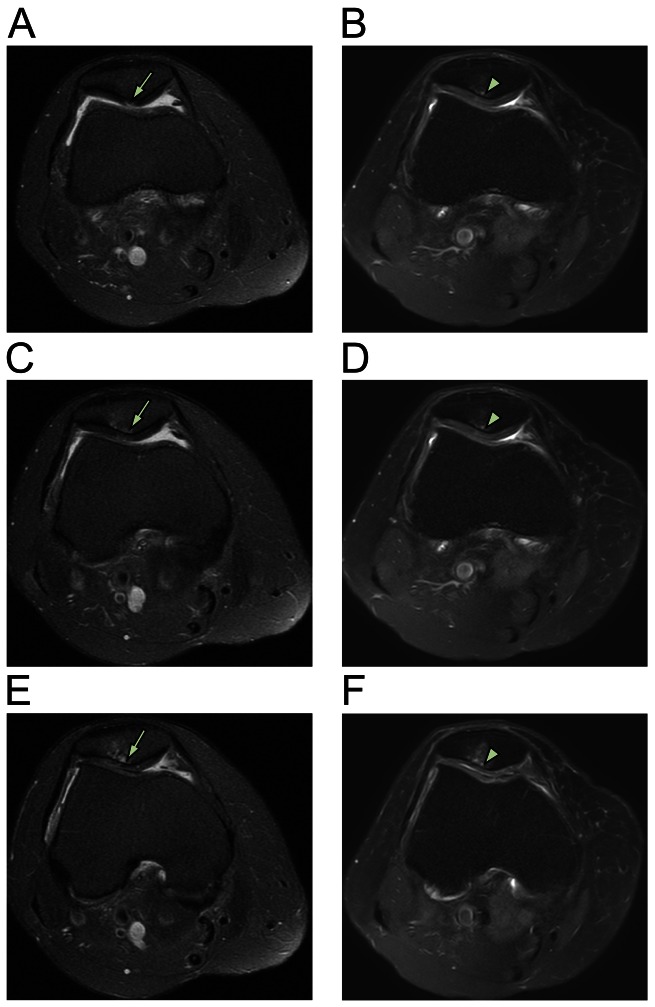
MRI axial sequential T2 views patient 3. Pre-treatment MRI scans of patient 3 (A [4/19], C [5/19], and E [6/19]) show retro-patellar signal changes (arrow) consistent with chondromalacia patellae along with medial meniscal maceration and cartilage thinning consistent with osteoarthritis. Post-treatment MRI scans at three months (B [5/20], D [6/20], and F [7/20]) show changes (arrowhead) consistent with probable cartilage restoration.

The patient #3 is a 63-year-old Korean female with more than 5 year history of right AKP. With the diagnosis of osteoarthritis, she had received one dose of steroid injection and multiple doses of PRP and hyaluronic acid injections over the last few years. However, she did not experience any improvement of AKP. The patient was seen by an orthopedic surgeon and was offered a total knee replacement (TKR). She was reluctant to go through TKR due to possible side effects. Since then, the patient had been receiving only PT without much improvement. At the time of initial evaluation in our clinic, the patient reported moderate AKP (VAS score: 5) on rest. AKP increased when walking (VWI: 8). The patient also complained of mild knee swelling. The FRI was 24 (Fig. 1C). On physical examination, there was mild medial joint tenderness with flexion and retro-patellar tenderness. Apley's and McMurray's tests were negative, and there was no ligament laxity. The Q-angle was 17. MRI scans showed retro-patellar signal changes consistent with CMP along with medial meniscal maceration and cartilage thinning consistent with osteoarthritis (Fig. 4A, C, and E).

### The follow-up disease surveillance questionnaires

All patients were followed up with telephone questionnaires every six months: at 6, 12, and 18 months. Each time, patients were asked the following questions: (i) did you experience the pain improvement due to the procedure? If no, please comment; (ii) did you experience any complications (i.e., infection, illness, etc.) you believe may be due to the procedure? If yes, please explain; and (iii) have you been diagnosed with any form of cancer since the procedure? If yes, please explain.

## Results

Two young patients (e.g.,<55 years), only with diagnosis of AKP, had failed conservative treatments comprising NSAIDs and PT. Their MRI scans of right knees showed retro-patellar signal changes consistent with CMP. Another patient, an elderly female with AKP and osteoarthritis, who failed conservative treatments consisting with multiple doses of PRP, hyaluronic acid, and/or steroid injections, was reluctant to go through a TKR due to possible side effects. The MRI scan showed retro-patellar signal changes consistent with CMP along with medial meniscal maceration and cartilage thinning consistent with osteoarthritis. Being wary of no non-invasive therapy for the cure of CMP, three patients wanted to try autologous, non-cultured ADSCs mixture-based treatments for the restoration of CMP. From June 2009, autologous, non-cultured ADSCs can now be used as a source of MSCs in Korea [5]. At that time, we obtained the informed consents from the patients.

After obtaining ADSCs by liposuction and preparing PRP, the ADSCs mixture was injected under ultrasonic guidance into the retro-patellar joints of all three patients on the day of liposuction. On the third, seventh, 14th, and 28th day after the initial injection, a different variation of PRP and CaCl_2_ with (or without) hyaluronic acid or a very low dose of dexamethasone (254.8 nmol/l) were injected in the same manner as that done in the first day. One month after the ADSCs mixture injection, each patient's pain improved 50–70% (Fig. 1A, B, and C). Three months after the treatment, the patients' pain improved 80–90% (Fig. 1A, B, and C). The repeated MRI scans at three months showed almost complete restoration of the damaged tissues (softened cartilages) on the patellae-femoral joints (panels B, D, and F of Figs. 2–4).

This is the first human report of a successful AKP reduction attributed to probable cartilage restoration by using percutaneous injections of the ADSCs mixture. As no non-surgical therapy is available for the cure of CMP, this study may significantly improve the current treatment strategy for young and old patients suffering from CMP with (or without) other joint diseases.

All three patients did not report any serious side effects. All patients experienced 2–3 days of joint discomfort after the first injection of ADSCs mixture. The discomfort was attributed to the volume expansion after the injection and completely disappeared after 2–3 days. Since ADSCs and PRP are autologous in nature, no rejection was expected and none occurred.

## Discussion

For diagnosis, MRIs of right knees were performed on three patients before ADSCs-mixture treatment. Consequently, post-treatment MRIs were repeated to compare pre- and post-treatment images. The MRI T2 sequence was used for its ability to differentiate bony anatomy from the cartilage. Due to slight differences in patient positioning and slight movement of patients during the MRI procedures, there was some difficulty in capturing the exact treatment locations. However, the pre-and post-treatment MRI results can be compared with sequential views to compensate for any possible errors [4].

In this study, significant MRI signal changes were apparent in the T2 views of right knees along the retro-patellar joint injection sites. These significant signal changes can be interpreted as possible signs of persistent and restored probable cartilages. Due to dramatic reduction in AKP, all three patients were reluctant to undergo knee cartilage biopsies to determine the true nature of the cartilage-like tissues. Although the true nature of the restored tissues is unclear, the damaged tissues (probable cartilages) have been believed to be restored, based on previous studies showing same tissue recovery using MSCs in patients with osteonecrosis, osteoarthritis, and meniscal injury [3], [4], [8]. The dramatic reduction in AKP and significant MRI signal changes without adverse effects support this notion.

With regard to the mechanism of the restoration, there are few plausible possibilities: (i) direct differentiation of the stem cells (e.g., ADSCs) [10]–[18]; (ii) trophic and paracrine effects of ADSCs on the existing cartilage [19]; (iii) effects of growth factors contained in PRP [20]; (iv) extracellular matrix production by chondrocytes stimulated by low physiologic doses of dexamethasone [21]; or (v) combination of all of the above-mentioned possibilities. Based on previous reports [3], [4], [8] and this study, ADSCs might play an important role in the restoration of the damaged tissues (softened cartilages). To our knowledge, this is the first human report of a successful restoration of CMP by using the new ADSCs mixture-based regimen.

Although the patients' symptoms and signs have improved, it is worthwhile to note that the improvement appeared gradually over three months. It can be postulated that three-months-time period is necessary for ADSCs to restore the softened cartilages.

It has been estimated that approximately 400,000 ADSCs are contained in 1 mL of adipose tissue [22]. Since 40 mL centrifuged adipose tissue were harvested, it is believed that approximately 16,000,000 stem cells were extracted and injected into knee joints.

These three clinical reports were not the randomized and controlled trial, and the key clinical feature in this study is performing the potentially effective and non-invasive treatment in the patients with continuous AKP and without history of trauma. Therefore, there is not a placebo (ADSCs-free) group. However, patient 3 had received PRP and hyaluronic acid injections without ADSCs over the last few years but she did not experience any improvement of AKP. She has experienced the dramatic reduction of AKP in CMP by percutaneously injection of ADSCs mixture. This clinical study was in compliance with the Declaration of Helsinki and regulation guidelines of KFDA (Korean Food and Drug Administration).

In conclusion, CMP of the knee is managed conservatively with NSAIDs and PT. When such conservative treatment fails, there is no definite cure for CMP thus presenting a major therapeutic challenge. However, this pilot study presents an alternative and safe way through which physicians may be able to manage CMP by percutaneous injections of autologous ADSCs mixture without culture expansion. Further studies with a larger number of patients with CMP are needed to confirm the short- and long-term efficacy and safety of ADSCs mixture-based treatment of CMP that are resistant to conventional therapies.
